# Functional Characterization of the HuR:CD83 mRNA Interaction

**DOI:** 10.1371/journal.pone.0023290

**Published:** 2011-08-04

**Authors:** Dorothea Pieper, Susann Schirmer, Alexander T. Prechtel, Ralph H. Kehlenbach, Joachim Hauber, Jan Chemnitz

**Affiliations:** 1 Department of Cell Biology and Virology, Heinrich Pette Institute - Leibniz Institute for Experimental Virology, Hamburg, Germany; 2 Zentrum für Biochemie und Molekulare Zellbiologie, Universität Göttingen, Göttingen, Germany; University of Cambridge, United Kingdom

## Abstract

Maturation of dendritic cells (DC) is characterized by expression of CD83, a surface protein that appears to be necessary for the effective activation of naïve T-cells and T-helper cells by DC. Lately it was shown that CD83 expression is regulated on the posttranscriptional level by interaction of the shuttle protein HuR with a novel posttranscriptional regulatory RNA element (PRE), which is located in the coding region of the CD83 transcript. Interestingly, this interaction commits the CD83 mRNA to efficient nuclear export via the CRM1 pathway. To date, however, the structural basis of this interaction, which potentially involves three distinct RNA recognition motifs (RRM1–3) in HuR and a complex three-pronged RNA stem-loop element in CD83 mRNA, has not been investigated in detail. In the present work we analyzed this interaction in vitro and in vivo using various HuR- and CD83 mRNA mutants. We are able to demonstrate that both, RRM1 and RRM2 are crucial for binding, whereas RRM3 as well as the HuR hinge region contributed only marginally to this protein∶RNA interaction. Furthermore, mutation of uridine rich patches within the PRE did not disturb HuR:CD83 mRNA complex formation while, in contrast, the deletion of specific PRE subfragments from the CD83 mRNA prevented HuR binding in vitro and in vivo. Interestingly, the observed inhibition of HuR binding to CD83 mRNA does not lead to a nuclear trapping of the transcript but rather redirected this transcript from the CRM1- towards the NXF1/TAP-specific nuclear export pathway. Thus, the presence of a functional PRE permits nucleocytoplasmic trafficking of the CD83 transcript via the CRM1 pathway.

## Introduction

Dendritic cells (DC) are the most potent antigen presenting cells (APC) of the immune system. Fully activated DC are able to induce T-cell mediated immunity and have the capacity to activate even naïve CD4^+^ or CD8^+^ T-cells [1], [2]. Therefore, DC are termed “nature's adjuvant”. Immature DC are able to take up and process antigens, followed by migration of the cells to the secondary lymphoid organs, where they mature. During this process a modulation of several expression profiles (chemokines and chemokine receptors), as well as up-regulation of several cytokines, costimulatory molecules and adhesion molecules occurs, that collectively promote DC∶T cell interaction and subsequently T-cell activation (reviewed in [3]–[5]).

Surface expression of CD83 protein serves as a marker for immune-competent mature DC [6], [7]. Although the exact function of CD83 remains to be investigated, various studies provided evidence that membrane-bound and soluble CD83 are important regulators of the immune system, for example affecting lymphocyte maturation as well as DC-mediated T-cell stimulation (reviewed in [7]–[10]).

Since membrane-bound CD83 is strongly upregulated during DC maturation and activation, it became of particular interest to investigate the regulation of CD83 expression in detail on both, the transcriptional and the posttranscriptional level [11], [12]. Interestingly, the latter investigation provided initial evidence that the transcript encoding CD83 is, in DC, exported from the nucleus via an uncommon route [12].

In metazoans, the vast majority of mRNAs are transported from the nuclear site of transcription to the cytoplasm, the site of protein synthesis, via the NXF1/TAP pathway (for reviews on nuclear mRNA export see [13], [14]). However, it has also been shown that the nucleocytoplasmic transport of a small subset of cellular transcripts, including the CD83 mRNA, is mediated by the unrelated CRM1 pathway [15]. CRM1 is the major nuclear export receptor that interacts with nuclear export signals (NESs) and mediates transport of a large variety of cellular proteins from the nucleus to the cytoplasm [16], [17]. A detailed analysis of the CD83 transcript revealed the presence of a highly-structured *cis*-active RNA element in the coding region, termed the posttranscriptional regulatory element (PRE), which is directly recognized by the cellular RNA-binding shuttle protein HuR [18].

The human HuR protein is a ubiquitously expressed member of a family of RNA-binding proteins that is related to the *Drosophila* ELAV (embryonic lethal abnormal vision) protein [19]–[22]. HuR appears to be a multifunctional regulator that is involved in the posttranscriptional processing of specific mRNA subsets by affecting their stability, transport or translation (reviewed in [23]–[25]. Binding of HuR to transcripts bearing U- and AU-rich RNA elements (AREs) [26], which are commonly located in their 3′-untranslated region (UTR), results in significant stabilization of these otherwise highly labile mRNAs (reviewed in [24], [27]. In contrast, binding of HuR to the coding sequence PRE of CD83 mRNA does not affect the stability of this transcript, but instead induces its efficient CRM1-mediated cytoplasmic accumulation [18]. Since HuR is unable to directly interact with CRM1 [28], both, PRE-bound HuR and CRM1 have to be connected by an adaptor molecule. In fact, functional analysis of the known HuR protein ligands pp32 (ANP32A) and APRIL (ANP32B) [28] revealed that the latter links HuR and CRM1 during nucleocytoplasmic translocation of CD83 mRNA in a highly specific manner [29], [30].

In the present work we have characterized the HuR:CD83 mRNA interaction by in vitro binding assays using various HuR and PRE RNA mutant constructs. Furthermore, we analyzed the contribution of the CRM1- and the NXF1/TAP-specific nuclear export pathway to the nucleocytoplasmic transport of selected CD83 mRNA mutants (i.e. PRE mutants) in transfected cell cultures.

## Results

### HuR binds specifically the PRE in CD83 mRNA

**Figure 1:**
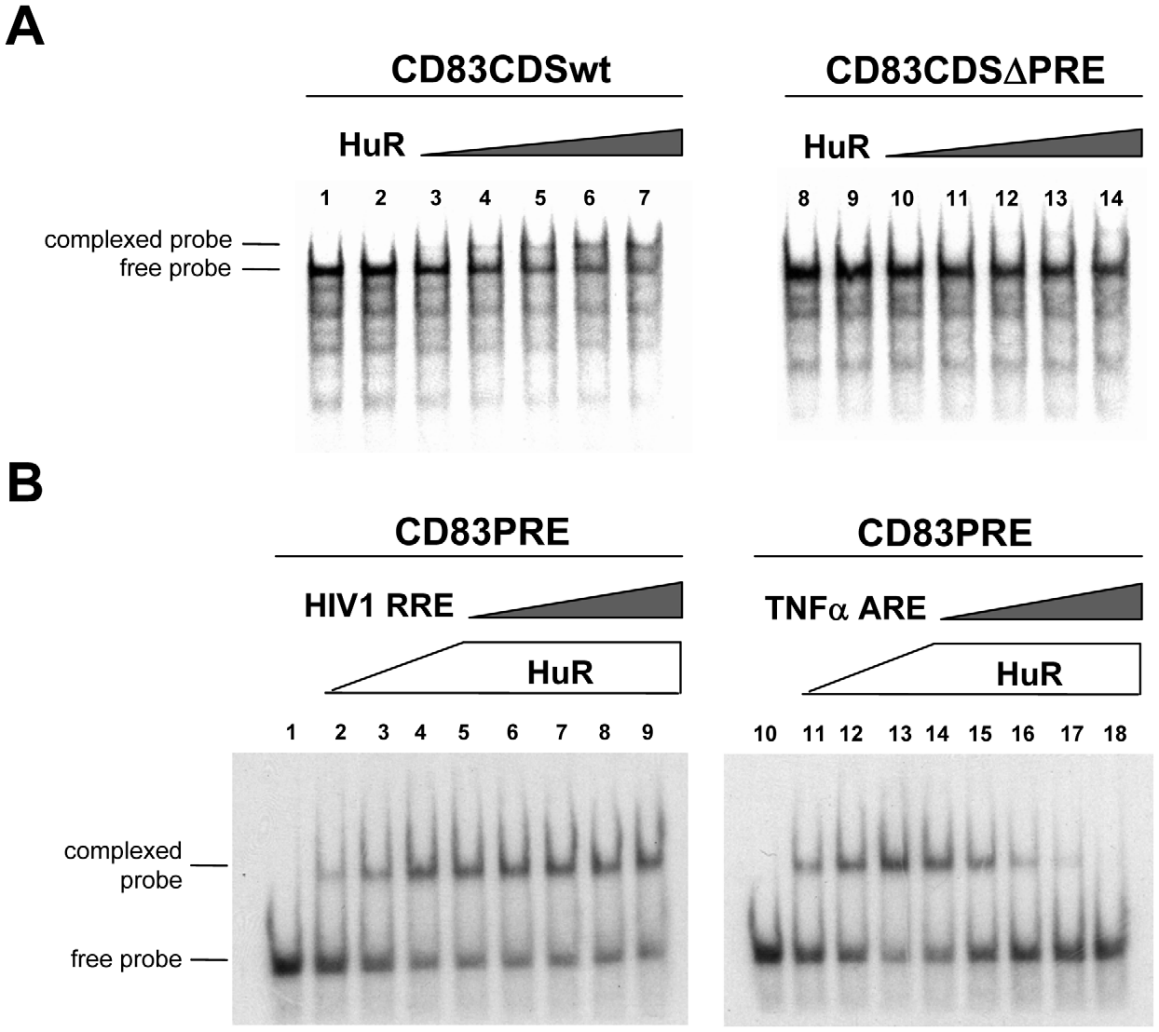
HuR binds in a specific manner to the CD83 PRE RNA region. **A.** Increasing amounts of bacterial expressed GST-HuR protein was incubated either with the radiolabelled CD83 mRNA wildtype (wt) coding sequence (CD83wt; lane 1–7) or the respective probe lacking the HuR target sequence PRE (CD83ΔPRE; lane 8–14). Complex formation was visualized by gel retardation assay and autoradiography. Lane 1: CD83wt RNA alone; lane 2: GST negative control; lane 3–7 increasing amounts of GST-HuR (0.095–0.475 µM); lane 8: CD83ΔPRE alone; lane 9: GST negative control; lane 10–14: increasing amounts of GST-HuR (0.095–0.475 µM). **B.** Analysis of PRE binding specificity by competition experiments. GST-HuR protein (lane 2–4 and lane 11–13: 0.119 µM, 0.238 µM, 0.475 µM, respectively; lane 5–9 and 14–18: 0.475 µM, respectively) or GST (1 µM) for negative control (lane 1 and 10) was incubated together with radiolabelled CD83wt PRE mRNA and analyzed as before. Increasing amounts (1–5 fold excess over CD83wt PRE mRNA) of either unlabelled HIV-1 RRE RNA (lane 5–9) or unlabelled TNFα ARE RNA (lane 14–18) were added to individual binding reactions.

To assess the binding of HuR to CD83 mRNA, RNA gel retardation experiments were performed. For this, HuR was expressed and purified in the context of a fusion to GST (glutathione S-transferase) and then analyzed in combination with in vitro transcribed radiolabelled CD83-specific RNA probes. In agreement with data presented in a previous study [18], addition of increasing amounts of GST-HuR to the CD83 wildtype (wt) coding region probe (nt 1–615) [31] resulted in the appearance of an RNA∶protein complex with slower mobility upon nondenaturing gel electrophoresis (Figure 1A, lane 3–7), compared with the migration of the uncomplexed RNA probe (Figure 1A, lane 1) or when GST alone was added to the binding reaction (Figure 1A, lane 2). In contrast, complex formation was not observed, even at high HuR concentrations, when the CD83 wt sequence was substituted by a corresponding RNA probe in which the PRE (nt 466–615) was deleted (CD83ΔPRE; see Figure 1A, lane 8–14). Thus, the PRE constitutes the only HuR target structure in the CD83 coding sequence.

Next, we investigated the specificity of this protein∶RNA interaction. Therefore, RNA competition experiments were performed in which increasing amounts of unlabelled and highly structured heterologous RNAs were added to the CD83 PRE:HuR binding reaction. As shown in Figure 1B, addition of the unrelated Rev Response Element (RRE) RNA, which constitutes the high-affinity binding site of the RNA-binding retroviral Rev transactivator in incompletely-spliced or unspliced HIV-1 transcripts [32], [33], did not negatively affect PRE:HuR complex formation (lane 5–9). In sharp contrast, however, strong binding competition, as indicated by diminished PRE-specific signals, was observed when the TNFα ARE, an established and prototypic HuR target sequence [34], [35], was used in these experiments (Figure 1B, lane 14–18). In sum these data indicated specific interaction of HuR with CD83 mRNA.

### RRM1 and RRM2 of HuR are necessary for CD83 mRNA binding

**Figure 2:**
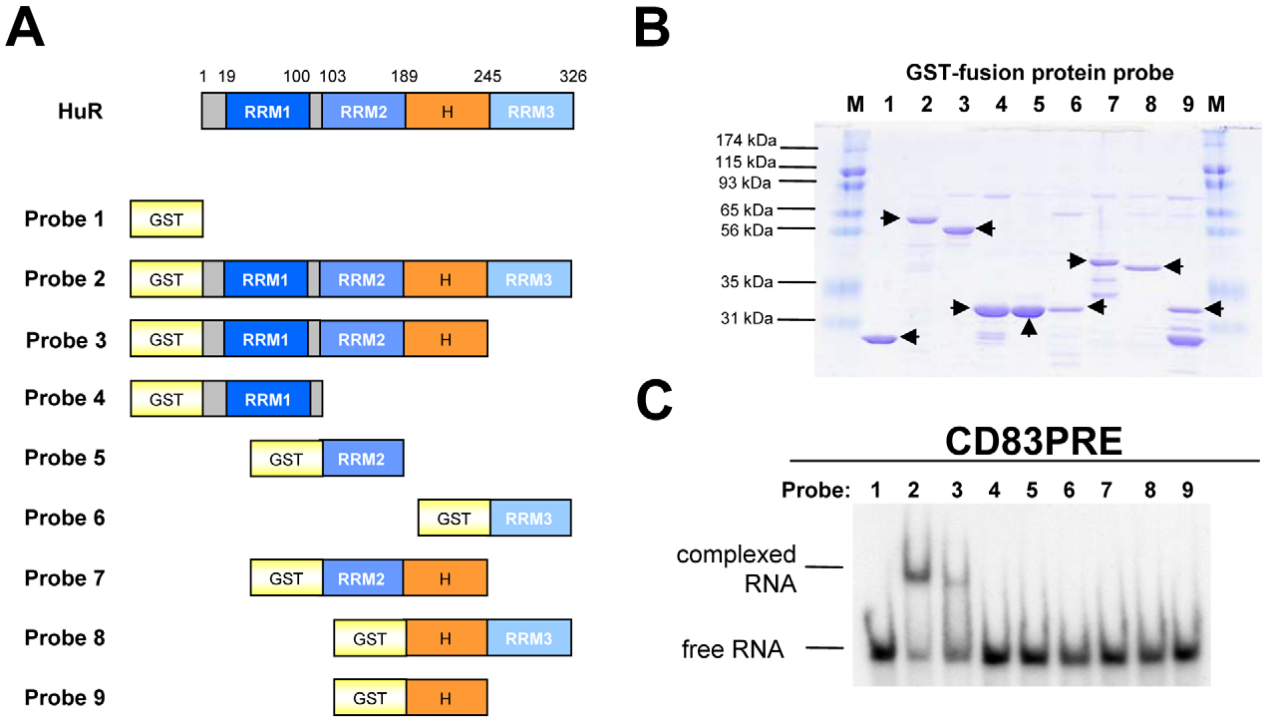
RRM1 and RRM2 of HuR are necessary for efficient CD83 mRNA recognition. **A.** Schematic diagram of the domain structure of HuR. The positions of the three RNA recognition motifs (RRM1–3) and the hinge region (H) in the human 326 amino acid (aa) HuR protein are indicated. Recombinant proteins (indicated by probe #) which were subsequently analyzed for CD83 PRE binding are depicted. Probe numbers correspond to gel lanes in panel B and C. **B.** Coomassie-stained SDS-PAGE of the recombinantly expressed and purified proteins (indicated by arrowheads). M, marker proteins. **C.** Radiolabelled CD83wt PRE coding sequence RNA was incubated either with GST (negative control) or with various HuR-derived GST-fusion proteins. Lane 1: GST; lane 2: full-length HuR; lane 3: HuR aa 1–244 (RRM1-RRM2-H); lane 4: HuR aa 27–93 (RRM1); lane 5: HuR aa 108–174 (RRM2); lane 6: HuR aa 246–317 (RRM3); lane 7: HuR aa 103–244 (RRM2-H); lane 8: HuR aa 190–328 (H-RRM3); HuR aa 175–245 (H). CD83 PRE RNA:protein interaction was analyzed by gel retardation assay as before.

HuR belongs to a family of ELAV-like proteins that are characterized by a modular structure composed of several functionally distinct domains [19], [22]. In particular, the interaction of HuR with RNA is mediated via three independent copies of the classical RNA recognition motif (RRM1–3), which is one of the most abundant protein domains in eukaryotes [36], [37]. While RRM1 and RRM2 are arranged in tandem, RRM2 and RRM3 are separated by a less conserved flexible hinge region (Figure 2A), which has been reported to contain a sequence required for nucleocytoplasmic shuttling of HuR [38].

To investigate which region of HuR is involved in CD83 mRNA binding, recombinant GST and several GST-HuR fusion proteins were prepared (Figure 2, A and B) and subjected to CD83 RNA-specific gel retardation assay as before (Figure 2C). Inspection of the obtained data revealed that a HuR mutant comprising RRM1, RRM2 and the hinge region clearly binds to the CD83 PRE sequence but, when compared to the full-length protein, with apparently reduced affinity (Figure 2C, lane 2 and 3). In contrast, RRM1 or RRM2 by itself failed to bind to the CD83 input RNA probe (Figure 2C, lane 4 and 5), a result that strongly resembled the data obtained when GST alone was used in a control experiment (Figure 2C, lane 1). In the same manner, neither RRM3 nor hinge domain-containing variants of RRM2 or RRM3 were able to interact with CD83 mRNA (Figure 2C, lane 6–8). As expected, also the GST fusion protein containing the isolated HuR hinge region failed to interact with the RNA probe in these experiments (Figure 2C, lane 9). These experiments therefore suggest that both, RRM1 and RRM2 are necessary for CD83 mRNA binding, whereas the more carboxyterminal regions of HuR seem not to be directly involved in PRE RNA target recognition.

### All potential CD83 PRE subloops contribute to HuR binding

**Figure 3:**
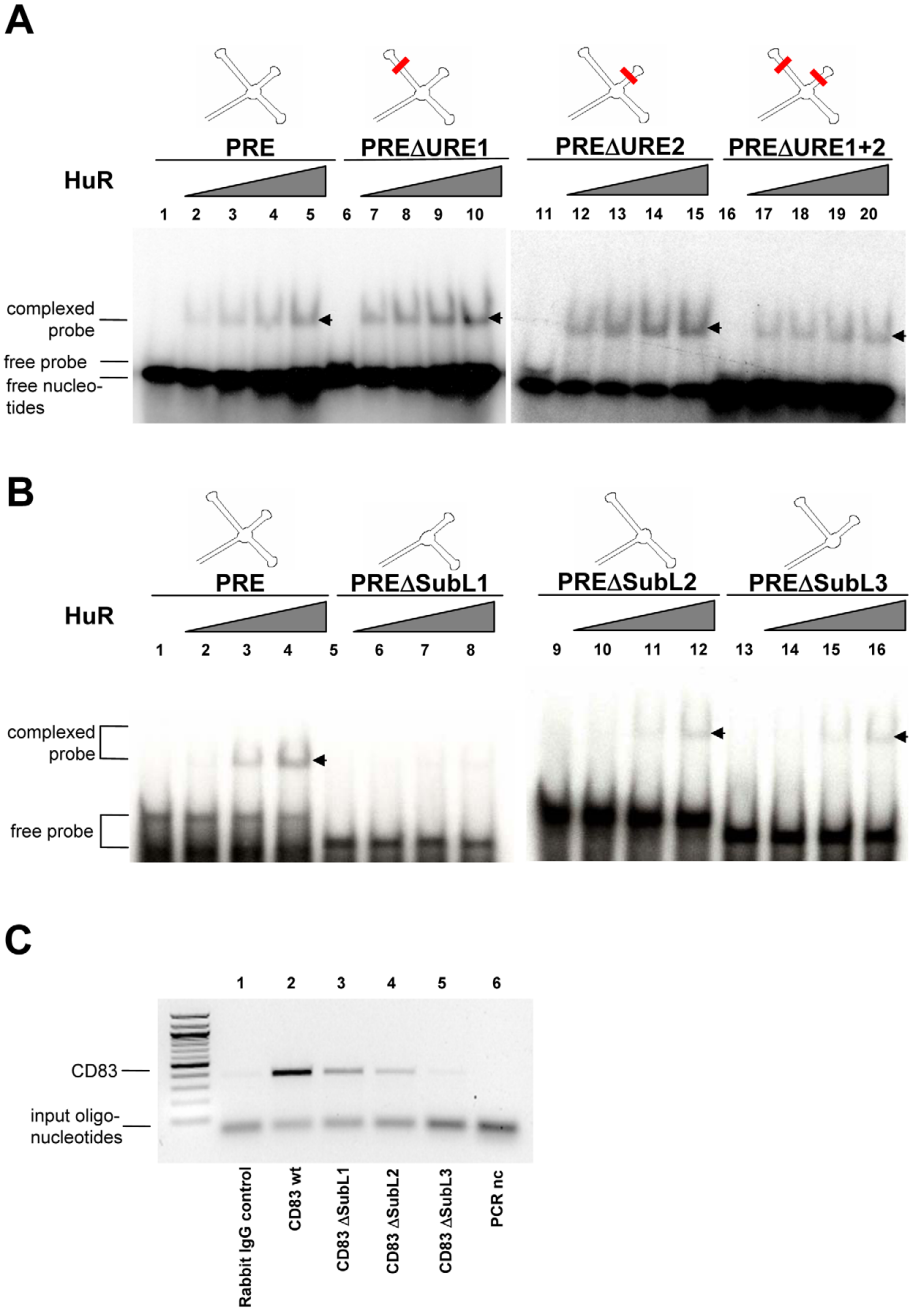
Deletion of individual CD83 PRE substructures impairs binding to HuR. **A.** GST alone (negative control, 2.2 µM, lane 1, 6, 11, 16) or increasing amounts of recombinant GST-HuR (0.19 µM, 0.38 µM, 0.57 µM, 0.95 µM) were incubated with radiolabelled CD83wt PRE RNA or several combinations of uridine-rich element (URE) mutants as indicated at the top of the panels. Formation of protein-RNA interaction (indicated by arrowhead) was subsequently detected by gel retardation analysis. **B.** GST alone (negative control; 2.2 µM, lane 1, 5, 9, 13) or increasing amounts of recombinant GST-HuR (0.237 µM, 0.475 µM, 0.95 µM; lanes 2–4, 6–8, 10–12, 14–16, respectively) were incubated together with radiolabelled full-length CD83wt PRE RNA or various PRE subloop (SubL) deletions (depicted at the top of the panels). Complex formation (indicated by arrowhead) was subsequently visualized by gel retardation assay as before. **C.** COS7 cells were transfected with expression vectors encoding for human CD83 cDNA flanked by the entire homologous 5′- and 3′-UTR (lane 1 and 2) or derivatives thereof (lane 3: ΔSubL1; lane 4: ΔSubL2; lane 5: ΔSubL3). Cellular lysates were subjected to immuno-PCR using anti-HuR antiserum (lane 2–5) or rabbit IgG for negative control (lane 1). CD83-specific RNA was detected by PCR followed by gel electrophoresis. A mock reaction without template served as additional negative control (nc; lane 6). This experiment has been reproduced at least three times with the same results.

Inspection of the PRE revealed, that this CD83 mRNA-derived sequence does not contain a classical ARE [18], [26]. However, two uridine-rich patches that are part of the PRE (UUUUUCU at nt 522–28 and UUUUCU at nt 549–54) could potentially serve as an HuR binding site (see [39] and references therein). To test this possibility, several CD83 PRE mutants were constructed in which these potential binding sites, termed URE1 and URE2 (uridine-rich elements 1 and 2; CUUCUCA and AUUCCU, respectively), were either individually or collectively altered by wobble mutagenesis (i.e. without affecting the coding capacity of the respective sequence). Gel retardation experiments using these constructs revealed that neither the mutation of a single URE (Figure 3A, lane 7–10 and lane 12–15), nor the alteration of both sequence elements (Figure 3A, lane 17–20) significantly affected the binding of HuR to the PRE. These data suggested that binding of HuR to CD83 mRNA represents not a simple sequence-specific association.

The CD83 PRE is a structured RNA element that potentially forms a stem loop with three apical substructures [18]. Hence, we next constructed a series of CD83 PRE mutants in which these apical substructures, termed subloop (SubL) 1 to 3, were individually deleted. The in vitro analysis of the respective RNAs by HuR-specific gel retardation assay indicated that deletion of SubL1 (ΔSubL1), corresponding to nucleotides (nt) 498–537 in the CD83 mRNA coding region [18], [31], abolished the binding of HuR to PRE RNA (Figure 3B, lane 2–4 versus lane 6–8). However, when SubL2 (nt 543–555) or SubL3 (nt 561–594) was deleted from the PRE, some residual binding was still observed (Figure 3B, lane 10–12 and 14–16, respectively). These in vitro data suggested that the entire CD83 PRE is necessary for efficient HuR binding.

To detect an association of HuR with CD83 mRNA in vivo, we next performed an immuno-PCR experiment. COS7 cells were transiently transfected with expression vectors encoding either for human CD83 cDNA flanked by the entire homologous 5′- and 3′-untranslated region (UTR) or variants thereof, encoding PRE SubL-deletions. At day two posttransfection, endogenous HuR protein was covalently crosslinked to the CD83 mRNA by addition of formaldehyde to the respective cell culture. Afterwards, HuR was immunoprecipitated using a polyclonal HuR antiserum, bound CD83 mRNA was reverse transcribed and the presence of coimmunoprecipitated CD83 mRNA was determined by specific PCR. By using this experimental set-up, it has been already previously shown that no specific binding could be observed when the CD83 message lacked all three PRE SubL elements [18]. In agreement with this data and shown in Figure 3C, the endogenous HuR protein strongly interacted with the full-length CD83 transcript (lane 2). In contrast, however, deletion of the individual SubL structures resulted in an intermediate HuR-binding phenotype, which was reflected by the diminished rescue of CD83-specific sequences (lane 3–5). Control experiments in which rabbit IgG (lane 1) was used for immunoprecipitation or in which the PCR template was omitted (lane 6) resulted in the expected negative phenotypes. Overall, these in vivo experiments not perfectly mirrored, but resembled to a significant extent the data obtained before by in vitro gel retardation assay. This indicates that the entire PRE contributes to HuR binding, although the data may also point to a more complex binding scenario which has not yet been fully understood.

### The PRE directs CD83 mRNA into the CRM1 nuclear export pathway

**Figure 4:**
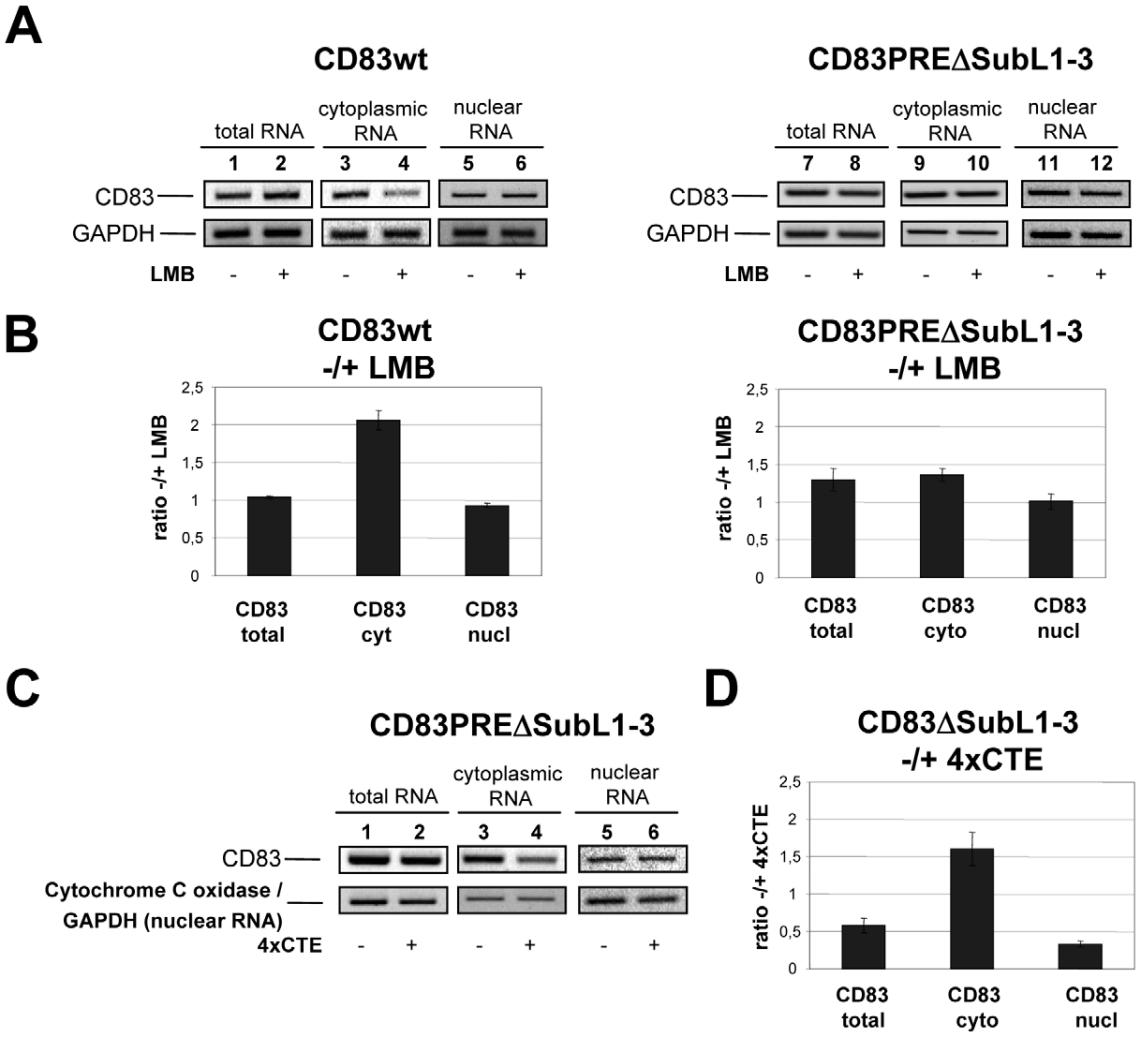
CRM1-mediated nuclear export of CD83 mRNA PRE. **A.** COS7 cells were transiently transfected either with an expression vector encoding for human CD83 cDNA flanked by the homologous 5′- and 3′-UTR (wt; lane 1–6) or with a related vector in which essential PRE sequences were deleted (PREΔSubL1–3; lane 7–12). At day two posttransfection cultures were exposed to 10 nM of the CRM1 inhibitor LMB or DMSO (solvent control) for 8 hours. Total, cytoplasmic and nuclear RNA was isolated, subjected to CD83- and GAPDH-specific (negative control) PCR and analyzed by gel electrophoresis. **B.** Quantitative real-time PCR of the RNA probes shown in panel A. RNA ratios +LMB/−LMB are depicted. **C.** Total, cytoplasmic and nuclear RNA was isolated from cell cultures which were cotransfected with the CD83 PREΔSubL1–3 expression vector and either a construct expressing four tandem repeats of the MPMV CTE (4×CTE) or the respective parental vector (negative control). For NXF1/TAP-independent control, mitochondrial cytochrome C oxidase mRNA was detected in total and cytoplasmic RNA, while GAPDH-specific transcripts were detected in nuclear RNA. **D.** Quantitative real-time PCR of the RNA probes shown in panel C. RNA ratios +/− NXF1/TAP inactivation (+CTE/−CTE) are depicted.

It has been previously shown that CD83 expression and the nuclear export of CD83 mRNA is sensitive to leptomycin B (LMB) [18], [40], a small-molecular weight compound that covalently modifies an essential cysteine residue in CRM1 and thereby inactivates selectively this nuclear export receptor [41], [42]. We therefore wanted to investigate the influence of the PRE subdomains with respect to the cellular mRNA nuclear export pathways. Therefore, COS7 cells were transfected with the CD83 expression vector as before. Two days after transfection, the cell culture was exposed to 10 nM LMB for 8 hours. Subsequently, total, cytoplasmic and nuclear RNA was isolated and reverse-transcribed into cDNA for PCR-mediated detection of CD83 and GAPDH (control) transcripts. Confirming previous studies [18], [40], the cytoplasmic accumulation of CD83 mRNA was significantly impaired when CRM1 activity was blocked by LMB in this experiment (Figure 4A, lane 3 and 4), whereas in the total and the nuclear RNA fraction no reduction of CD83 mRNA levels was observed (Figure 4A, lane 1 and 2 and lane 5 and 6, respectively). By following this experimental design we next analyzed a CD83 transcript in which PRE SubL1–3 was deleted (ΔSubL1–3). In sharp contrast to the results described above, LMB did now not affect the cytoplasmic accumulation of this CD83 mRNA, suggesting that CRM1 was not operational in the nuclear export of PRE SubL1–3-deficient transcripts (Figure 4A, lane 7–12). To further substantiate this observation, we subjected the respective RNAs also to quantitative real-time PCR. This confirmed the notion, that the PRE in CD83 mRNA permits nuclear export of this transcript via the CRM1 pathway (Figure 4B).

Since the PRE-deficient CD83 transcript was apparently exported from the nucleus in a CRM1-independent fashion (Figure 4A and B), we now wanted to investigate the potential involvement of an alternative, CRM1-unrelated export route in this transport process. Clearly, the export receptor NXF1/TAP, which is known to transport the bulk of cellular transcripts out of the nucleus [13], [14], is a prime candidate to specify such a pathway. Unfortunately, to date no small-molecular weight inhibitor that specifically targets NXF1/TAP exists. However, the “simple” D-type retrovirus Mason-Pfizer monkey virus (MPMV) exploits the NXF1/TAP-pathway by directly binding NXF1/TAP via a structured RNA sequence element, termed the constitutive transport element (CTE) [43]. Therefore, intracellular overexpression of multiple CTE NXF1/TAP target sites provides a means to inhibit NXF1/TAP activity. As expected and shown in Figure 4C, overexpression of four tandem repeats of the MPMV CTE (4×CTE) [44] did indeed negatively affect the nuclear export of CD83 PREΔSubL1–3 mRNA (lane 3 and 4), reflecting a functionally compromised NXF1/TAP pathway. Again, this result was quantified by real-time PCR (Figure 4D).

Obviously, the PRE in CD83 mRNA enables nucleocytoplasmic translocation of this mRNA via the CRM1-pathway, whereas deletion of the PRE makes this RNA subject to regulation by the NXF1/TAP export receptor and its associated factors.

## Discussion

The CD83 protein serves as surface marker for fully matured DC [45], [46]. Although its exact function is still unknown [47], various independent findings suggest that this protein plays a pivotal role in immune regulation and may therefore hold great potential for therapeutic application [6]–[9].

Lately it was shown that the expression of CD83 is regulated at the level of nucleocytoplasmic transport [15], [18]. Generally, the translocation of the vast bulk of cellular mRNAs across the nuclear envelope is mediated by the nuclear export factor NXF1/TAP [13], [14]. In comparison, nuclear mRNA export mediated by the importin β-related export receptor CRM1 appears to be a rather rare event, since CRM1 usually mediates the transit of noncoding rRNA and U snRNA, ribosomal subunits and numerous proteins through the nuclear pore complex [17], [48]. However, CRM1 is exploited by HIV-1 to export the viral unspliced and incompletely-spliced mRNAs [49]. Interestingly, by employing a systematic screen in human cells, several cellular mRNAs were identified that are also exported from the nucleus to the cytoplasm via the CRM1 pathway [15]. These transcripts are expressed upon cellular activation or differentiation and include the transcript encoding the CD83 molecule [15], [18]. Association of CRM1 with the CD83 transcript requires specific adaptor molecules. In particular, the RNA-binding cellular shuttle protein HuR has been identified to directly bind with high-affinity the *cis*-active CD83 PRE mRNA sequence [18]. Subsequently, CRM1 is recruited to the nuclear CD83 mRNA:HuR ribonucleoprotein (RNP) complex by APRIL (ANP32B) [29], a family member of a group of leucine-rich acidic nuclear phosphoproteins that is able to bind at the same time to both, HuR and CRM1 [28]. Thus, initial recognition of the CD83 transcript by HuR is pivotal to all consecutive processes that eventually result in CRM1-mediated nuclear export of HuR-bound CD83 transcripts.

The RNA-binding HuR protein has been shown to efficiently shuttle between the nuclear and cytoplasmic compartment [50], [51]. It appears that both, its RNA-binding and nucleocytoplasmic shuttling activity can be attributed to distinct domains of HuR [19], [22], [38]. Upon nuclear import, HuR binds via its RRMs to specific mRNAs. These mostly belong to the class of short-lived early response gene (ERG) mRNAs that encode functionally important proteins such as protooncoproteins, cytokines and lymphokines. Their intrinsic instability depends on the presence of ARE sequences [26], which enhance deadenylation rates as well as mRNA degradation [52]–[54]. Importantly, upon binding to HuR these ARE-containing transcripts are profoundly stabilized [50], [52,52].

It has been demonstrated that ARE recognition by HuR is mediated by the protein's two tandemly arranged RRMs (RRM1 and RRM2) [39], [55], while the carboxyterminal RRM3 appears to bind to the poly(A) tail of the respective ERG mRNA [56]. Deletion of RRM3 in the closely related HuD protein has been shown to only moderately reduce its ARE binding affinity [57]. Moreover, it has also been reported that the RRM3 in HuR does not contribute to high affinity binding, but is rather required for the cooperative assembly of HuR oligomers on RNA [58]. These phenotypes closely resemble the HuR binding-data raised in the present study, indicating that RRM1 and RRM2 are essential for efficient recognition of the CD83 PRE RNA sequence and that RRM3 is not directly involved in PRE interaction (Figure 2C). However, binding of HuR to the ARE [55], as well as to the PRE (Figure 2C), occurred most efficiently when the RRM3 was present, despite the fact that the in vitro analyzed RNAs were devoid of a poly(A) tail, as mentioned before, constituting an established RRM3 binding substrate [56]. Thus, in case of both HuR target sequences, the ARE and the PRE, highly efficient protein∶RNA complex formation appears to require the presence of all three RRMs. This may suggest that the tandemly arranged RRM1 and RRM2 primarily provide binding specificity to HuR, while RRM3 further stabilizes this binding event by secondary interactions with the respective RNA target.

The RNA binding and immuno-PCR experiments performed in the present study demonstrated that all PRE substructures are required for optimal HuR binding (Figure 3). Furthermore, these data confirmed in an independent approach the previous notion that the CD83 PRE:HuR interaction is not solely based on the association of HuR with uridine-rich patches, but rather depends on HuR's recognition of the complete PRE structure [18]. Interestingly, deletion of the PRE from the CD83 mRNA directed this transcript away from the CRM1- into the NXF1/TAP-specific nuclear export pathway (Figure 4). Thus, the *cis*-active PRE secures nucleocytoplasmic CD83 mRNA translocation via CRM1. One may therefore speculate that this specific export route might provide a significant advantage with respect to efficient protein synthesis when compared to the more general pathway involving NXF1/TAP, particularly under conditions of cellular activation. In fact, this notion is increasingly supported by studies identifying specific cellular transcripts that are translocated from the nucleus to the cytoplasm by CRM1. These include the transcript coding for interferon-α1, c-Fos, Cyclin D1, cyclooxygenase-2, HLA-A and, most notably, HuR itself [28], [59]–[64]. An additional example is represented by the κ-opioid receptor (KOR) mRNA that also utilizes HuR and CRM1 to form a nuclear export complex [65], thereby resembling the mechanism involved in the nuclear export of CD83 mRNA. Moreover, in analogy with the CD83 PRE a *cis*-active element has been recently identified in the coding region of interferon-α1 mRNA that mediates, although in an HuR-independent manner, the aforementioned CRM1-dependent nuclear export of this message [66]. Taken together, these data suggest that utilization of CRM1, either via HuR and its ligands (e.g. APRIL/ANP32B), or via HuR-unrelated adaptors, is frequently a common denominator of mRNAs that are induced during cell activation or differentiation. Conceivably, this mechanism ensures the timely and efficient nuclear export, and thereby protein expression, of these transcripts.

## Materials and Methods

### Molecular Clones

The plasmids p3CD83-CDS (CD83 coding sequence [CDS] nucleotides [nt] 1–615; [31], p3CD83-PRE (nt 466–615), p3TNFα-ARE, pGEM-RRE, pGex-HuR (amino acid [aa] 1–328; full length HuR) and p3UTR-CD83 were published previously [18]. The vector p4×CTE expresses 4 tandem copies of the MPMV CTE [44] which were ligated between EcoRI and XhoI sites of pcDNA3 (Invitrogen). For in vitro transcription, the plasmids p3CD83CDSΔPRE (CD83 CDS deletion nt position 466–615), p3CD83PREΔSubL1 (CD83 CDS deletion nt position 498–537), p3CD83PREΔSubL2 (CD83 CDS deletion nt position 543–555) and p3CD83PREΔSubL3 (CD83 CDS deletion nt position 561–594), p3CD83PREΔURE1 (nt position 522–528, GAT TTT TCT→GAC TTC TCA), p3CD83PREΔURE2 (nt position 549–554, GCT TTT CTC→GCA TTC CTA) and p3CD83PREΔURE1+2 (nt position 522–528, GAT TTT TCT→GAC TTC TCA; nt position 549–554, GCT TTT CTC→GCA TTC CTA), were constructed using PCR technology. The respective CD83 CDS-derived fragments were ligated between the HindIII and EcoRI sites of the pcDNA3 vector.

Likewise, the eukaryotic expression vectors p3UTR-CD83ΔSubL1 (CD83 CDS nt 498–537 deletion), p3UTR-CD83ΔSubL2 (CD83 CDS nt 543–555 deletion), p3UTR-CD83ΔSubL3 (CD83 CDS nt 561–594 deletion), p3UTR-CD83ΔSubL1–3 (CD83 CDS nt 498–594 deletion), were constructed by ligating the respective PCR-generated CD83 CDS-derived fragments between the HindIII and Asp718 sites of the p3UTR-CD83 vector [18].

The expression plasmids for purification of the GST-proteins, pGex-HuR1–244 (aa 1–244; RRM1 + RRM2 + hinge region), pGex-HuR27–93 (aa 27–93; RRM1), pGex-HuR108–174 (aa 108–174; RRM2), pGex-HuR246–317 (aa 246–317; RRM3), pGex-HuR103–244 (aa 103–244; RRM2 + hinge region), pGex-HuR190–328 (aa 190–328; hinge region + RRM3) and pGex-HuR175–245 (aa 175–245; hinge region) are based on the parental vector pGex-5×-1 (Pharmacia Biotech).

### Protein Purifications and RNA Gel Retardation Assays

GST fusion proteins were expressed in *E. coli* BL21 and purified from crude lysates by affinity chromatography on glutathione-Sepharose 4B (Pharmacia) as described previously [67]. RNA gel-retardation assays were performed using in vitro-transcribed [^32^P]-labelled CD83-derived probes, MS2 competitor RNA and GST fusion protein as previously described [68], except that RNA-protein complexes were separated on 4% or 6% native polyacrylamide gels.

### In Vitro Transcription

Labelled and non-labelled RNA was obtained by in vitro transcription with or without [α-^32^P] UTP (Amersham Pharmacia) using a commercial T7 RNA polymerase-based kit following the manufacturer's instructions (Promega).

### Immuno-PCR

COS7 cells (5×10^6^) were transfected with 8 µg DNA of either p3UTR-CD83, p3UTR-CD83ΔSubL1, p3UTR-CD83ΔSubL2 or p3UTR-CD83ΔSubL3 expression vector, respectively, using the DEAE dextran transfection method. Two days after transfection endogenous HuR protein was covalently cross-linked to CD83 mRNA by adding 1% formaldehyde to the medium for 5 minutes. Afterwards, crude extracts were prepared and HuR was immunoprecipitated with a polyclonal HuR antiserum, bound CD83 mRNA was reverse transcribed and the presence of coimmunoprecipitated CD83 mRNA sequences was determined by specific PCR using DNA-oligos described below. Rabbit IgG (instead of HuR antibodies) or water was used for PCR control.

### Cell Culture and Transfections

The cell line COS7 (ATCC CRL-1651™) was maintained as previously described [69]. For analysis of CD83 RNA-synthesis and distribution 2.5×10^5^ COS7 cells were transfected with 250 ng of either p3UTR-CD83 or p3UTR-CD83ΔSubL1–3 vector, using DEAE-dextran and chloroquine as previously described [18]. Likewise, 1 µg of the p4×CTE expression vector was cotransfected with 250 ng of either the p3UTR-CD83 or p3UTR-CD83ΔSubL1–3 vector, respectively.

### RNA Isolation and PCR Analyses

Total cellular RNAs were isolated according to the manufacturer's protocol by using TRIzol reagent (Invitrogen). For isolation of cytoplasmic and nuclear RNAs, 2×10^5^ cells were lysed on ice for 1 min using 100 µl NP40 buffer (10 mM Hepes- KOH pH 7.8, 10 mM KCl, 20% glycerol, 1 mM DTT, 0.25% NP40). Subsequently, the lysates were cleared by centrifugation at 470× *g* for 5 min at 4°C. Cytoplasmic RNA was isolated from 80 µl of the supernatant using TRIzol reagent. The nuclei were washed again in 100 µl NP40 buffer to deplete residual cytoplasmic RNA. Afterwards, the nuclear RNA was isolated by using TRIzol reagent. DNAse treatment of the RNA samples was performed. Selected RNA samples were reversely transcribed using the first strand cDNA (AMV) synthesis kit for RT-PCR (Roche Molecular Biochemicals) according to the manufacturer's instructions. Subsequently, RT products were assayed by PCR. For detection of GAPDH sequences following primers were used: forward, 5′-TGAAGGTCGGAGTCAACGGATTTGGT-3′; reverse, 5′-CATGTGGGCCATGAGGTCCACCAC-3′. The amplification profile involved 25 cycles of denaturation at 95°C for 1 minute, primer annealing at 56°C for 1 minute, and primer extension at 72°C for 2 minutes. CD83 mRNAs were detected by using following primer pairs: forward, 5′ GGT GAA GGT GGC TTG CTC CGA AG-3′, and reverse, 5′-GAG CCA GCA GCA GGA CAA TCT CC-3′. The amplification profile involved 25 cycles of denaturation at 95°C for 1 minute, primer annealing at 56°C for 1 minute, and primer extension at 72°C for 1 minute. Cytocrome C oxidase subunit II mRNAs were detected by using following primer pairs: forward, 5′ GCA AGT AGG TCT ACA AGA CG-3′, and reverse, 5′-GTA GTC GGT GTA CTC GTA GG-3′. The amplification profile involved 25 cycles of denaturation at 95°C for 1 minute, primer annealing at 56°C for 1 minute, and primer extension at 72°C for 1 minute. Real time PCR was performed as described previously [18].
